# Supplementary material for: Factors associated with postoperative visual function after rhegmatogenous retinal detachment with foveal detachment
Source: PLoS One. 2024 Sep 20;19(9):e0308863. doi: 10.1371/journal.pone.0308863 (PMC11414927; doi:10.1371/journal.pone.0308863)
Supplement: Attachment — Submitted filename: Response_to_Reviewers_20240702.docx [file pone.0308863.s002.docx]

July 2^nd^, 2024

Kogo Jiro

Academic Editor

*PLOS ONE*

Re: Revised manuscript, PONE-D-24-04377R1

Title: Factors associated with postoperative visual function after rhegmatogenous retinal detachment with foveal detachment

Dear Dr. Kogo:

It is a pleasure to communicate with you again in relation to our abovementioned manuscript, which underwent a major revision. We have tried to amend the manuscript in accordance with the specific and general requests, and we provide below a point-by-point response to your and the reviewers’ comments. All changes in our revised manuscript are indicated by red font.

We thank you and the reviewers for your thoughtful suggestions and insights, from which our manuscript has greatly benefited. We look forward to working with you and the reviewers to move this manuscript closer to publication in *PLOS ONE.*

Once again, I take this opportunity to thank you, the editors, and the reviewers who we believe did an excellent editorial review job that has increased the scientific merit of this paper. Please note that we remain open to further requests for changes from you and the reviewers.

Thank you for your consideration. I look forward to hearing from you and hope to work with *PLOS ONE* again in the future.

Sincerely,

Sho Yokoyama

Department of Ophthalmology, Japan Community Healthcare Organization Chukyo Hospital

1-1-10 Sanjo Minami-ku Nagoya-city, Aichi prefecture, Japan

E-mail: yokoyama@chukyogroup.jp

Reviewer #2: Authors have addressed the previous reviewers’ comments well and consequently the manuscript has been revised well. However, I still find several points to be revised further mainly in the parts that have been changed by revision.

We thank the reviewer for the thoughtful comments that have increased the scientific value of our paper. We have carefully revised the manuscript based on the reviewer’s suggestions.

***Comment 1:***

1. Abstract: “Conclusions” are written in a style like a copy of the results. Please revise the conclusions to summarize the results briefly in one or two sentences.

**Response 1:**

Thank you for this pertinent suggestion. The Conclusions section of the Abstract has been revised to provide a summary of our results.

Conclusions in Abstract:

“Our study findings suggest that BCVA, metamorphopsia, and retinal sensitivity at 1 year after vitrectomy for RRD with foveal detachment are influenced by distinct factors.” (lines 38–39)

***Comment 2:***

Statistical analysis: Lines 171-173 ‘including….. at 1 year postoperatively’ could be omitted because they duplicate with lines 176-177.

**Response 2:**

Thank you for the valuable advice. We removed the duplicated sentences and revised them.

Statistical analysis:

“Initially, univariate linear regression analysis was performed to estimate the relationships of pre-, intra-, and postoperative factors with postoperative visual function. Subsequently, multivariate linear regression analyses were conducted using the stepwise method, incorporating factors with P-values <0.1 in the univariate linear regression analysis as explanatory variables and BCVA, vertical and horizontal metamorphopsia degree determined using M-CHARTS, and retinal sensitivity determined using the MP-3 at 1 year postoperatively as the objective variables.” (lines 164–170)

***Comment 3:***

Results; Multiple regression analysis: I would like to request authors to revise whole sentences in an organized way. For example, lines 197-201 should be omitted because the same thing has been described in the methods; for each of BCVA, vertical and horizontal metamorphopsia, descriptions of ‘significantly associated with’ and ‘which was associated with significantly worse …’ should be presented in a single and simple sentence to avoid duplications; lines 204-207 might be ‘ILM peeling and longer duration were associated with higher vertical metamorphopsia score’, not ‘metamorphopsia was associated with ILM peeling and longer duration’; same in lines 208-211; ‘1 year postoperatively’ appears too often; etc.

**Response 3:**

Thank you for these valuable comments, and we agree with the reviewer’s suggestions. The Results (both in the Abstract and the main manuscript text) were revised accordingly.

Results in the Abstract:

“Preoperative total retinal detachment was the only factor significantly associated with worse BCVA at 1 year postoperatively (β=0.589, P<0.001). Intraoperative ILM peeling (β=0.443, P=0.003) and longer duration after recognizing visual dysfunction (β=0.425, P=0.005) were significantly associated with higher vertical metamorphopsia scores at 1 year. The horizontal metamorphopsia score was significantly related to the duration after recognizing visual dysfunction (β=0.457, P=0.008). The disappearance of the EZ line on OCT at 3 months postoperatively (β=−0.638, P<0.001) was significantly associated with lower retinal sensitivity at 1 year.” (lines 30–37)

Multiple regression analysis:

“The results of the multiple regression analysis are presented in Table 3. Preoperative total retinal detachment was the only factor significantly associated with worse BCVA at 1 year postoperatively (β=0.589, P<0.001). For vertical metamorphopsia at 1 year, intraoperative ILM peeling (β=0.443, P=0.003) and longer duration after recognizing visual dysfunction (β=0.425, P=0.005) were significantly associated with higher scores. The horizontal metamorphopsia score was significantly related to the duration after recognizing visual dysfunction (β=0.457, P=0.008), indicating that a longer time to surgery was associated with worse scores for both vertical and horizontal metamorphopsia. Additionally, the disappearance of the EZ line on OCT at 3 months postoperatively (β=−0.638, P<0.001) was significantly associated with lower retinal sensitivity at 1 year.” (lines 191–200)

***Comment 4:***

1st paragraph of discussion: Usually discussions start with a summary of the results in the current study, whereas the present writing of this paragraph sounds like an introduction to the report by Park et al. Please revise this paragraph to focus on the authors’ own results and compare them with previous literature. It might be recommended to create a new paragraph as the 1st to describe the total results briefly and move on to the 2nd in which factors related to postsurgical visual acuity are discussed.

**Response 4:**

Thank you for this valuable suggestion. We fully agree with the reviewer’s comment and created a new first paragraph to summarize all study results.

Discussion:

“In this study investigating factors related to visual function at 1 year postoperatively, we found that preoperative total retinal detachment was significantly associated with worse BCVA after surgery. This result partially supports the findings of a previous study by Park et al. Intraoperative ILM peeling and longer duration after recognizing visual dysfunction were significantly associated with higher vertical metamorphopsia scores, and the horizontal metamorphopsia score was significantly related to the duration after recognizing visual dysfunction. Additionally, the disappearance of the EZ line on OCT at 3 months postoperatively was significantly associated with lower retinal sensitivity.” (lines 223–230)

“In the current study, total detachment emerged as a significant factor related to BCVA at 1 year postoperatively, echoing the findings by Park et al. that preoperative predictive factors include the extent of detachment.” (lines239–242)

***Comment 5:***

Reference 18 by Abdullah et al: in this paper, all eyes were treated using both PFCL and SO, which may have had some or significant impact on the retinal function and macular morphology particularly in the ILM-peeled area as has been suggested by some previous studies and meta-analysis including the one assessing metamorphopsia and discontinuity of the ORLs between eyes treated with and without PFCL. When authors compare their results with previous literature, those difference in the details of surgical procedures that may potentially affect the results should be carefully discussed.

**Response 5:**

Thank you for these valuable remarks. We agree with the reviewer’s suggestions. As you pointed out, in the report by Abdullah et al., PFCL was used intraoperatively, and they performed SO tamponade in all patients. We have added to the manuscript that these points need to be taken into consideration, as their surgical technique differs from that of our current study, and the use of PFCL and SO tamponade may have affected both macular structure and function postoperatively.

Discussion:

Conversely, Abdullah et al. evaluated macular structure and function with and without ILM peeling for RRD with foveal detachment using OCT angiography and multifocal electroretinography, revealing potential damage to macular structure and function in the ILM peeling group [18]. However, in the study by Abdullah et al., perfluorocarbon liquids (PFCLs) and silicone oil (SO) tamponade were used in all patients. Previous reports have indicated that the use of PFCLs is associated with increased discontinuity in the interdigitation zone following retinal detachment surgery compared with not using PFCLs [19]. Additionally, SO tamponade has been associated with postoperative thinning of both the inner and outer retinal layers compared with gas tamponade [20]. Therefore, it is important to consider the potential effects of PFCLs and SO tamponade on macular structure and function.” (lines 257–267)

19.	McKay BR, Bansal A, Kryshtalskyj M, Wong DT, Berger AR, Muni RH. Two-year outcomes of different subretinal fluid drainage techniques during vitrectomy for fovea-off rhegmatogenous retinal detachments: ELLIPSOID-2 Study. Br J Ophthalmol. 2024 (online ahead of print). https://doi.org/10.1136/bjo-2023-323879.

20.	Huang RS, Mihalache A, Lau THA, Popovic MM, Kertes PJ, Muni RH. Pars plana vitrectomy with silicone oil or gas tamponade for uncomplicated retinal detachment: a systematic review and meta-analysis. Am J Ophthalmol. 2024;266: 144-155. https://doi.org/10.1016/j.ajo.2024.05.008.

***Comment 6:***

Discussion, lines 274-281: authors discuss that the discontinuity of Ez lines was recovered in many cases from 3 months to 1 year, while the Ez status at 3 months was predictive of retinal sensitivity at 1 year. To me, these two descriptions do not fit each other well and it is not clear how they are connected to each other in the structure of the paragraph.

**Response 6:**

Thank you for pointing this out. We have added a sentence summarizing the two statements.

Discussion:

Retinal sensitivity measurements demonstrated an association with the morphology of the EZ line at 3 months postoperatively, implying that patients in whom the EZ line in the central fovea disappeared at 3 months postoperatively might experience reduced retinal sensitivity within the central 20° macular area at 1 year postoperatively. The EZ line at 3 months postoperatively was continuous in 5 patients, discontinuous in 24 patients, and absent in 4 patients. In contrast, the EZ line at 1 year postoperatively was continuous in 22 patients and discontinuous in 11 patients, with no case of disappearance, indicating an improvement in the EZ line over time after surgery. Thus, the initial state of the EZ line at 3 months is important for predicting long-term retinal sensitivity, although many patients experience recovery of the EZ line from 3 months to 1 year postoperatively.” (lines 275–284)

***Comment 7:***

In the newly added paragraph in Discussion part (from the line 296), some points are discussed very well regarding the discrepancy in postoperative metamorphopsia in comparison with previous reports. However, the first point proposed by the authors (preoperative RRD status and symptom duration) may need to show some trend that those status were not typical (if any) among their patients compared to other studies.

**Response 7:**

Thank you for your remarkable comments. In the cited article, Yamada et al. [22] reported a period from subjective symptoms to surgery of 5.25±6.37 days, which is shorter than that in the present study (8.59±21.01 days), and fewer than two quadrants were affected by retinal detachment in 27 of 33 cases, suggesting that the preoperative retinal detachment status may have been milder in their study than in the present study. In addition, the preoperative retinal detachment status was not reported by Borowicz et al. (2019) and Okuda et al. (2018). We evaluate these points in the Discussion section.

Discussion:

“First, the preoperative retinal detachment status and the duration of foveal detachment might have varied between previous studies and the current study. Yamada et al. [22] reported a period from subjective symptoms to surgery of 5.25±6.37 days, which is shorter than the 8.59±21.01 days observed in our study. Moreover, the retinal detachment area affected fewer than two quadrants in 27 out of 33 cases in Yamada et al., suggesting that the preoperative retinal detachment status in their study might have been milder than that in the current study. Additionally, the number of quadrants with retinal detachment and the duration of foveal detachment were not specified in Borowicz et al. [21] and Okuda et al. [23]. Therefore, the recovery over time might have differed among these four studies depending on the retinal detachment status of the patients.” (lines 304–313)

***Comment 8:***

The last point in the same paragraph to note the difference in the fluid drainage techniques (lines 310-312): If this aspect affected the results, it could be interpreted that drainage from the original break using endoscopy had negative impact on the postoperative recovery of metamorphopsia and retinal sensitivity. Is it true? Personally, I do not think so because results presented in some previous literatures showed better outcomes after drainage from the original break than drainage from posterior retinotomy site, although I do not know if there is any previous report to show advantage or disadvantage when endoscopy is utilized.

**Response 8:**

Thank you for your valuable feedback. The use of an endoscope allows for the drainage of subretinal fluid through existing retinal tears without the use of PFCL or the creation of a drainage retinotomy. As the reviewer pointed out, these points can result in favorable postoperative outcomes. Although no previous studies have demonstrated the usefulness of subretinal fluid drainage using an endoscope, it has been reported that avoiding the creation of a drainage retinotomy can improve the success rate of RRD surgery and reduce the incidence of postoperative ERM (Ohara et al., 2022; Ishikawa et al., 2022). Other reports implicated the use of PFCLs in postoperative visual acuity reduction and disruption of the outer retinal structures (Kumari et al., 2022). Citing these references, we have added the possibility that differences in surgical methods between previous publications and our current study may have favored the early recovery of macular function in our study.

Discussion:

“It has been reported that avoiding the creation of a retinotomy during the drainage of subretinal fluid can improve the success rate of RRD surgery and reduce the incidence of postoperative ERM [24,25]. Additionally, the use of PFCLs has been associated with a reduction in postoperative visual acuity and the disruption of the outer retinal structures [26]. These differences in surgical methods may have favored the early recovery of macular function in the present study, and at 3 months postoperatively, macular function recovery was nearly complete, with no further recovery observed over time.” (lines 321–327)

24.	Ohara H, Yuasa Y, Harada Y, Hiyama T, Sadahide A, Minamoto A, et al. Drainage retinotomy is a risk factor for surgical failure after pars plana vitrectomy in patients with primary uncomplicated rhegmatogenous retinal detachment. Retina. 2022;42: 2307-2314. https://doi.org/10.1097/iae.0000000000003608.

25.	Ishikawa K, Akiyama M, Mori K, Nakama T, Notomi S, Nakao S, et al. Drainage retinotomy confers risk of epiretinal membrane formation after vitrectomy for rhegmatogenous retinal detachment repair. Am J Ophthalmol. 2022;234: 20-27. https://doi.org/10.1016/j.ajo.2021.07.028.

26.	Kumari N, Surve A, Kumar V, Azad SV, Chawla R, Venkatesh P, et al. Comparative evaluation of outcomes of drainage techniques in vitrectomy for rhegmatogenous retinal detachment. Retina. 2022;42: 27-32. https://doi.org/10.1097/iae.0000000000003259.
