# Supplementary material for: Factors associated with postoperative visual function after rhegmatogenous retinal detachment with foveal detachment
Source: PLoS One. 2024 Sep 20;19(9):e0308863. doi: 10.1371/journal.pone.0308863 (PMC11414927; doi:10.1371/journal.pone.0308863)
Supplement: Attachment — Submitted filename: Response_to_Reviewers_20240730.docx [file pone.0308863.s003.docx]

July 30^th^, 2024

Kogo Jiro

Academic Editor

*PLOS ONE*

Re: Revised manuscript, PONE-D-24-04377R2

Title: Factors associated with postoperative visual function after rhegmatogenous retinal detachment with foveal detachment

Dear Dr. Kogo:

It is a pleasure to communicate with you again in relation to our abovementioned manuscript, which underwent a minor revision. We have tried to amend the manuscript in accordance with the specific and general requests, and we provide below a point-by-point response to your and the reviewers’ comments. All changes in our revised manuscript are indicated by red font.

We thank you and the reviewers for your thoughtful suggestions and insights, from which our manuscript has greatly benefited. We look forward to working with you and the reviewers to move this manuscript closer to publication in *PLOS ONE.*

Once again, I take this opportunity to thank you, the editors, and the reviewers who we believe did an excellent editorial review job that has increased the scientific merit of this paper. Please note that we remain open to further requests for changes from you and the reviewers.

Thank you for your consideration. I look forward to hearing from you and hope to work with *PLOS ONE* again in the future.

Sincerely,

Sho Yokoyama

Department of Ophthalmology, Japan Community Healthcare Organization Chukyo Hospital

1-1-10 Sanjo Minami-ku Nagoya-city, Aichi prefecture, Japan

E-mail: yokoyama@chukyogroup.jp

Reviewer #2: Authors have addressed the previous reviewers’ comments well and consequently the manuscript has been revised well. However, I still find several points to be revised further mainly in the parts that have been changed by revision.

We thank the reviewer for the thoughtful comments that have increased the scientific value of our paper. We have carefully revised the manuscript based on the reviewer’s suggestions.

***Comment 1:***

The first paragraph of the Discussion is very long and does not focus on a single theme. As pointed out in the previous review, please present a brief summary of the current study in the first paragraph (never go beyond it), and then discuss each of the details in comparison with previous literatures in the following paragraphs. Please stick to the rule of writing to focus on one particular topic in each paragraph.

Response 1:

Thank you for your insightful suggestion. We have revised the first paragraph of the Discussion section as the reviewer recommended. The first paragraph now provides a summary of the current study, while the subsequent paragraphs discuss each detail in comparison with previous literature.

“In this study investigating factors related to visual function at 1-year postoperatively, we found that preoperative total retinal detachment was significantly associated with worse BCVA after surgery. Intraoperative ILM peeling and longer duration after recognizing visual dysfunction were significantly associated with higher vertical metamorphopsia scores, and the horizontal metamorphopsia score was significantly related to the duration after recognizing visual dysfunction. Additionally, the disappearance of the EZ line on OCT at 3 months postoperatively was significantly associated with lower retinal sensitivity.” (lines 224-230)

***Comment 2:***

Lines 242-243: In which study were “duration after recognizing visual dysfunction and the postoperative status of the EZ line not identified as associated factors? In the current study, those factors were shown to be associated with visual outcomes as illustrated in Table 3, then were they not identified in Park’s report? Please note this point clearly. This confusion occurs partly due to the inappropriate configuration of the paragraphs as suggested above.

Response 2:

Thank you for your question.

In our study, we did not identify “duration after recognizing visual dysfunction and the postoperative status of the EZ line” as factors associated with BCVA in the first postoperative year. On the other hand, Park et al. identified the extent of detachment; duration of macula-off; preoperative external limiting membrane (ELM) integrity; and postoperative outer retinal microstructures, particularly the photoreceptor outer segment layer and postoperative microstructure of the outer retina, as factors associated with visual acuity in the first year. We have revised the text as follows to clarify the differences between this study and previous reports, including Park et al.

“Park et al. examined factors influencing visual acuity 1-year postoperatively in 180 eyes undergoing surgery for RRD with foveal involvement. They reported that predictive factors included the extent of detachment, duration of macula-off, preoperative external limiting membrane (ELM) integrity, and postoperative outer retinal microstructures, particularly the photoreceptor outer segment layer [6]. Additionally, previous research has emphasized the significance of OCT morphology after RRD surgery, indicating that the continuity of the postoperative EZ and ELM lines, as well as the presence of a foveal bulge, correlate with final BCVA after RRD surgery [12–14].

On the other hand, our study identified total detachment as a significant factor related to BCVA 1-year postoperatively, aligning with Park et al.'s findings that preoperative predictive factors include the extent of detachment. However, our study did not identify the duration after recognizing visual dysfunction or the postoperative status of the EZ line as associated factors. The discrepancy between previous reports of the duration of macula-off and the OCT morphology after RRD surgery being associated with postoperative visual acuity and our results may be attributed to the relatively small sample size in our study and the less stringent evaluation of the duration of foveal detachment.” (lines 231-247)

***Comment 3:***

Lines 260-270: The newly added sentences (lines 260-267; seems too long and interferes the flow of the discussion) and the next sentence (“The findings of the current study also indicate that ….”) do not logically connected to each other smoothly. The structure of discussion should be re-considered for this paragraph.27–32. doi: 10.1097/iae.0000000000003259.

Response 3:

Thank you for your valuable suggestion. We have made the following changes to the structure of the text in the Discussion section.

“The duration after recognizing visual dysfunction was identified as an associated factor for both vertical and horizontal metamorphopsia scores assessed using M-CHARTS. Although the duration after recognizing visual dysfunction may not always coincide with the period of foveal detachment, these two intervals are considered similar owing to the impact of central foveal detachment on visual acuity. Thereby, the current study results suggest that postoperative metamorphopsia may worsen with a longer duration since the patient acknowledged central vision dysfunction.

Additionally, intraoperative ILM peeling was associated with worse postoperative 1-year vertical metamorphopsia scores. In the context of vitrectomy for patients with RRD, ILM peeling may be performed to prevent postoperative ERMs [15,16]. In regard to postoperative retinal function with ILM peeling in RRD cases, Pietras-Trzpiel et al. reported no significant difference in postoperative metamorphopsia scores assessed using M-CHARTS between patients with or without ILM peeling during vitrectomy for RRD [17]. Conversely, Abdullah et al. evaluated macular structure and function with and without ILM peeling for RRD with foveal detachment using OCT angiography and multifocal electroretinography, revealing potential damage to macular structure and function in the ILM peeling group [18]. However, in that study, perfluorocarbon liquids (PFCLs) and silicone oil (SO) tamponade were used in all patients. Reportedly, the use of PFCLs is associated with increased discontinuity in the interdigitation zone following RRD surgery compared with that when not using PFCLs [19]. Additionally, SO tamponade has been associated with postoperative thinning of both the inner and outer retinal layers compared with that using gas tamponade [20]. Therefore, it is important to consider the potential effects of PFCLs and SO tamponade on macular structure and function.

In the current study, the decision to perform ILM peeling was left to the discretion of individual surgeons, and it is possible that more severe cases were more likely to undergo ILM peeling. Indeed, the higher the preoperative PVR grade, the more frequently ILM peeling was performed. However, the results of multiple regression analysis, which included the presence of ILM peeling and PVR grade as factors, showed that PVR grade was not related to postoperative visual function, including visual acuity, metamorphopsia, and retinal sensitivity. In summary, the current study suggests that ILM peeling may impact retinal function and exacerbate metamorphopsia regardless of the severity of the PVR grades.“ (lines 248-277)
